# Fer and FerT Govern Mitochondrial Susceptibility to Metformin and Hypoxic Stress in Colon and Lung Carcinoma Cells

**DOI:** 10.3390/cells10010097

**Published:** 2021-01-07

**Authors:** Odeya Marciano, Linoy Mehazri, Sally Shpungin, Alexander Varvak, Eldad Zacksenhaus, Uri Nir

**Affiliations:** 1 The Mina and Everard Goodman Faculty of Life-Sciences, Bar-Ilan University, Ramat-Gan 5290002, Israel; Odeya.martsiano@biu.ac.il (O.M.); mahazrl@biu.ac.il (L.M.); Sally.Shpungin@mail.biu.ac.il (S.S.); avarvak@biu.ac.il (A.V.); 2 Laboratory of Medicine & Pathology, University of Toronto, Toronto, ON M5G 2M1, Canada; eldad.zacksenhaus@utoronto.ca

**Keywords:** Fer, FerT, reprogrammed mitochondria, oxidative phosphorylation, metformin, hypoxia, malignant cells

## Abstract

Aerobic glycolysis is an important metabolic adaptation of cancer cells. However, there is growing evidence that reprogrammed mitochondria also play an important metabolic role in metastatic dissemination. Two constituents of the reprogrammed mitochondria of cancer cells are the intracellular tyrosine kinase Fer and its cancer- and sperm-specific variant, FerT. Here, we show that Fer and FerT control mitochondrial susceptibility to therapeutic and hypoxic stress in metastatic colon (SW620) and non-small cell lung cancer (NSCLC-H1299) cells. Fer- and FerT-deficient SW620 and H1299 cells (SW∆Fer/FerT and H∆Fer/FerT cells, respectively) become highly sensitive to metformin treatment and to hypoxia under glucose-restrictive conditions. Metformin impaired mitochondrial functioning that was accompanied by ATP deficiency and robust death in SW∆Fer/FerT and H∆Fer/FerT cells compared to the parental SW620 and H1299 cells. Notably, selective knockout of the *fer* gene without affecting FerT expression reduced sensitivity to metformin and hypoxia seen in SW∆Fer/FerT cells. Thus, Fer and FerT modulate the mitochondrial susceptibility of metastatic cancer cells to hypoxia and metformin. Targeting Fer/FerT may therefore provide a novel anticancer treatment by efficient, selective, and more versatile disruption of mitochondrial function in malignant cells.

## 1. Introduction

Metastasis is the main cause of death among cancer patients, and targeting the metastatic process is therefore one of the major challenges in current cancer therapy. Increasing evidence suggests that migrating cancer cells and primary tumor cells utilize distinct metabolic pathways [1,2,3,4]. For instance, analysis of gene expression signatures in an orthotopic breast cancer model indicated that circulating tumor cells are enriched in factors that regulate mitochondrial respiration and biogenesis compared with primary and metastatic lesions [2]. Furthermore, the mitochondria of cancer cells are reprogrammed and modified compared to normal cells. For example, the mitochondrial glutamine uptake machinery which propels the tricarboxylic acid (TCA) cycle is upregulated in cancer cells [5,6]. Notably, other functional pathways including the electron transport chain (ETC) are also altered in the mitochondria of malignant cells. Specifically, complex I (Comp. I) mutations which could serve as metabolic determinants of malignant-cell sensitivities to glucose limitation are frequently observed in many cancers [7]. Two constituents that associate with the reprogrammed Comp. I in malignant but not normal somatic cells are the intracellular tyrosine kinase Fer and its sperm- and cancer-specific truncated variant, FerT [8,9]. Fer bears a kinase domain residing in its *C*-terminal part that is preceded by an SH2 domain and a 414 aa long *N* terminal tail bearing three coiled-coil domains and an FCH (Fps/Fes/Fer/CIP4 Homology) motif [10,11,12]. The genomic *Fer* locus is located on chromosome 5q21 [13] and contains two genes: *fer* and *ferT*, which encode two distinct kinases: Fer and FerT. An intronic promotor in the *fer* gene directs the expression of the FerT protein, which consequently gains a unique 43 aa long *N* terminal tail [14]. While Fer is expressed in all somatic cells except for pre-T, pre-B, and naïve T cells [15], the expression of FerT is normally restricted to spermatocytes, spermatids, and sperm cells [8,16]. However, FerT is also found in various subcellular compartments of malignant cells, and together with Fer, it associates with Comp. I of the mitochondrial ETC only in spermatogenic and cancer cells [8]. Fer was shown to regulate breast cancer cell adhesion, migration, and anoikis resistance and to be necessary for tumor growth and metastasis in mice [11,16,17,18,19]. Unlike Fer, not much is known about the regulatory roles of FerT in cancer cells. A better understanding of the roles of Fer and FerT in metabolic reprogramming of malignant cells may open new avenues for efficiently and selectively targeting the reprogrammed mitochondria of metastatic cells. 

Metformin, a guanidine derivative initially extracted from the plant *Galega afficinalis* (French lilac), has been used as a glucose-lowering medication in humans for more than 60 years [20]. Metformin exerts its primary effect at the molecular level as an inhibitor of oxidative phosphorylation (Oxphos) by reversibly inhibiting NADH dehydrogenase (mitochondrial ETC- Comp. I) activity, resulting in reduced ATP production [21,22,23]. The AMP-activated protein kinase (AMPK) is also a key molecular mediator through which metformin exerts its anticancer effects [24]. A meta-analysis on diabetic cancer patients treated with metformin reported a significant reduction in mortalities for various cancers [25,26,27]. These findings motivated the inclusion of metformin in numerous anticancer therapeutic combinations [28,29]. However, it turned out that the efficacy and therapeutic impact of metformin depends on the site and type of cancer [30]. Furthermore, it was shown that cancer cells, which are insensitive to low glucose supplementation, are also moderately sensitive to metformin treatment [7]. Thus, there is a profound importance in further unraveling regulatory factors that control the moderate susceptibility of cancer cells to metformin therapy. Since metformin targets the reprogrammed mitochondrial ETC of malignant cells, we sought to decipher the roles of Fer and FerT in modulating the susceptibility of cancer cell’s mitochondria to metformin-evoked stress. In this study, we show that Fer and its cancer-specific variant, FerT, are novel regulators of mitochondria vulnerability to mitochondrial stresses like metformin treatment and onset of hypoxic conditions.

## 2. Materials and Methods

### 2.1. Tissue Culture and Metformin Treatment

Colon cancer cell lines (HCT116, SW48, and SW480) and metastatic colon (SW620) cells were grown in Dulbecco’s modified Eagle’s medium (DMEM) (Gibco-Cat. 41965039). Non-small cells lung cancer (NSCLC; H1299) cells were grown in Roswell Park Memorial Institute (RPMI) medium (01-100-1A, Biological Industries (BI)). The mediums were supplemented with 10% fetal bovine serum (FBS, BI, 04-127-1A) and 5% Penicillin-Streptomycin-Nystatin (PSN, BI, 03-050-1A). Forty-eight hours prior to all experiments, cells were grown in glucose-deprived medium (DMEM without glucose, BI, 01-057-1A) supplemented with 2 mM l-glutamine (BI, 03-020-1B). Metformin (Millipore, 317240-5GM) was dissolved in ultrapure water to a 100-mM stock concentration. Metformin was diluted in cells growth media to the desired concentration as specified for the indicated time.

### 2.2. Generating SW/HΔFer/FerT and SW/HΔFer Cells

Knockout of the *fer* and *ferT* genes by the CRISPR-Cas9 paired nickases system was carried out according to the manufacturer’s instructions (Sigma) using the Cas9 D10A mutant fused to GFP [31] and a pair of gRNAs targeting a common region of Fer and FerT in Exon 12—gRNA1: TATTCTGGGAATTGCACCATGG and gRNA2: GAGAGAGTCATGGGAAACCTGG—or a pair of gRNAs targeting a specific region of Fer in Exon 6—gRNA1: GCTTTGTCGTATCGTTCCTTGG and gRNA2: TTGCACAATCAGTATGTATTGG. SW620 and H1299 cells were transfected with three plasmids using Lipofectamine 2000 (Invitrogen-Cat. 11668019) according to the manufacturer’s guides. Cells expressing the GFP-Cas9 were sorted by fluorescence-activated cell sorting (FACS)- FACSAriaIII (Becton Dickinson Biosciences, San Jose, CA 95131, USA). Authentication of the parental SW620 and H1299 cells, and all their derived clones was performed at the Genomic Center of Biomedical Core Facility, the Technion, Israel. All cell lines were authenticated using short tandem repeat (STR) profiling (please see the Supplementary Materials) within the last three years, and all experiments were performed with mycoplasma-free cells.

### 2.3. Subjecting Cells to Hypoxia

SW620, SWΔFer/FerT, SWΔFer, H1299, HΔFer/FerT, and HΔFer cells were grown for 48 h in glucose reach medium or in glucose-deprived medium (DMEM without glucose, Biological Industries) supplemented with 2 mM l-glutamine at 37 °C with 5% CO_2_. The cells were subjected to anaerobic culture jar containing CO_2_-generating envelope (GasPak EZ, BD Biosciences, BD260001) for an additional 24 h. These conditions reduced the oxygen level in the jar to 1% within 30 min.

### 2.4. Immunoblot Analysis

Whole-cell lysates were prepared as described before [8]. In brief, 30 μg protein lysates from each sample were resolved by 10% SDS–PAGE and analyzed by western blotting using polyclonal anti-Fer/FerT antibodies selectively directed toward the common SH2 domain of the two proteins [8]. Reacting protein bands were visualized using a horseradish peroxidase (HRP)-conjugated secondary antibody to rabbit or mouse IgG (Jackson,111-035-144, 115-035-062, respectively) in conjunction with a western blot (WB) chemiluminescence reagent (Pierce Cat. 34080).

### 2.5. Cell Death Analysis

Cells (5 × 10^5^) were seeded in 6-cm cell culture dishes in glucose-deprived medium (DMEM without glucose, BI, 01-057-1A) supplemented with 2 mM l-glutamine for 48 h. Cells were then treated with metformin at the indicated concentration for 24 h. Cells were stained with annexin V-FITC and propidium iodide (PI) using Annexin V-FITC Apoptosis Detection Kit (Biovision Cat. K101-100) following the manufacturer’s instructions. Staining was quantified by FACS ARIAIII. All data were analyzed using FlowJo software (FlowJo LLC, Ashland, Oregon, USA).

### 2.6. Quantification of Cellular ATP and NAD^+^ Levels

Cells were suspended in 0.5-mL cold perchloric acid (PCA) solution in 1.5-mL tubes. The mixture was incubated on ice for 15 min and was then centrifuged at 13,000× *g* for 2 min to remove the precipitate. The supernatant was neutralized followed by incubation with NaOH for 15 min on ice. After another centrifugation at 10,000× *g* for 2 min, the supernatants were taken for chromatographic analysis as described before [8].

### 2.7. Determination of Mitochondrial Activity

Oxygen consumption rate (OCR) was monitored as an indicator for mitochondrial respiration activity and was measured with an XF24 Extracellular Flux Analyzer using XF Cell Mito Stress Test kit according to the manufacturer’s instructions (Seahorse, XF Cell Mito Stress Test Kit, 103015-100, Agilent, Santa Clara, CA 95051, USA). Cell seeding number was optimized to 100,000 cells/well for SW620 cells. For determining mitochondrial susceptibility to stress cues, cells were plated into XF24 plates in glucose-deprived media for 48 h followed by metformin treatment (5 mM) for 16 h. For determining mitochondrial basal rate activities, mitochondrial respiratory chain drugs were added, following the Mito Stress kit specifications. One micromole of oligomycin was used to block ATP-linked oxygen consumption, 1 µM of carbonilcyanide p-triflouromethoxyphenylhydrazone (FCCP) was used as an uncoupling agent to obtain maximal respiration, and 0.5 µM of rotenone/antimycin A was applied to inhibit complexes I and III, thereby arresting all mitochondrial respiration. OCR was measured 3 consecutive times following the injection of each drug and was normalized to protein content.

### 2.8. Statistical Analysis 

Statistical analysis was performed using the paired and unpaired Student’s *t*-tests, with *p* < 0.05 being considered significant. The results are depicted as mean ± standard error (±SE) of the mean for n given samples.

For statistical analysis, percent change values were divided by 100 and log-transformed. Mean percent change of treatments and/or cell type were compared to the control group using one-sample *t*-tests against a mean of 0, and correction for multiple testing was applied using the false discovery rate (FDR) procedure. Treatments and/or cell types were compared using one-way or two-way ANOVA tests, followed by Tukey’s post hoc analysis. The normality of residuals assumption was assessed with residuals plots.

## 3. Results

### 3.1. Fer/FerT Deficiency Exacerbates Susceptibility of SW620 and H1299 Cells to Metformin 

**Figure 1:**
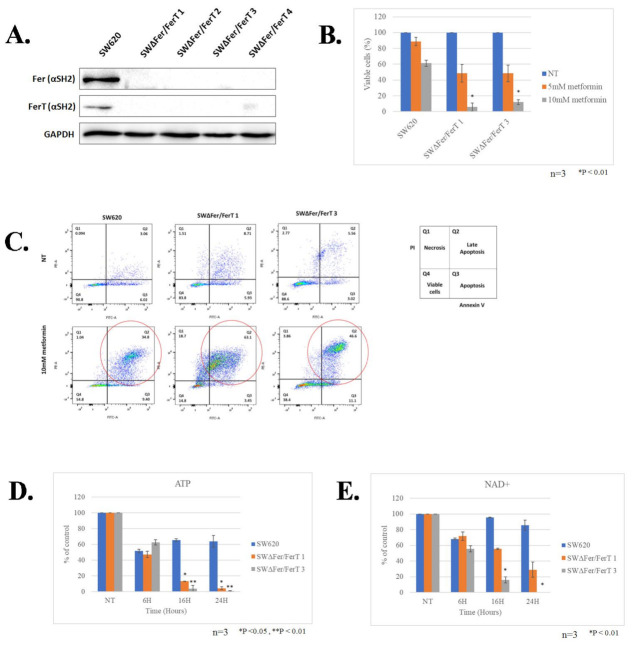
Lack of Fer and FerT renders metastatic colon cancer cells susceptible to metformin treatment: (**A**) Fer- and FerT-deficient SWΔFer/FerT cells were generated using the CRISPR-Cas9 system. Parental SW620 cells expressed both Fer and FerT and four derived SWΔFer/FerT knock-out clones. GAPDH served as a loading control. (**B**) Parental SW620 and SWΔFer/FerT cells were either left untreated (NT) or treated with 5-mM or 10-mM metformin for 24 h. Dead and viable cells were differentiated using Trypan Blue staining and were counted using the Countess II cell-counter. Values represent means ± SE (*n* = 3). (**C**) Metformin-treated or untreated cells were stained with annexin V/propidium iodide (PI) followed by flow-cytometry analysis. Annexin V staining (Q3) indicates apoptotic cell death. PI staining (Q1) indicates necrotic cell death. Annexin V/PI double staining (Q2) indicates late apoptotic/early necrotic cell death. This experiment represents one out of three independent experiments that gave similar results. An increase in late-apoptotic cell death after metformin treatment is marked with red circles (Q2). (**D**,**E**) ATP and NAD^+^ levels were decreased in metformin-treated SWΔFer/FerT cells. SW620 and SWΔFer/FerT grown in glutamine-supplemented glucose-deprived medium were left untreated (NT) or treated with 5-mM metformin for the indicated time periods: (**D**) cellular ATP levels and (**E**) cellular NAD^+^ levels. Values represent means ± SE. This experiment represents one out of three independent experiments that gave similar results.

To decipher the roles of Fer and FerT in modulating mitochondria susceptibility to stress cues in cancer cells, we initially focused on metastatic SW620 colon cancer (CC) cells, which express both Fer and FerT [16] (Figure 1A). We generated Fer- and FerT-deficient SW620 cells (SWΔFer/FerT) using the modified CRISPR-Cas9 mutated knockout system [31,32]. Expression analysis of Fer and FerT revealed efficient knockout of the *fer* and *fer*T genes in 4 SWΔFer/FerT clones (Figure 1A); SWΔFer/FerT clones #1 and #3 were selected for further studies.

To test the impact of Fer and FerT absence on the sensitivity of SW620 cells to the mitochondrial targeting effect of metformin, SWΔFer/FerT cells were cultured in a medium devoid of glucose and supplemented with glutamine. Under these conditions, glycolysis was halted and mitochondrial Oxphos became the main energy-generation process. While parental SW620 cells were only mildly affected by either 5- or 10-mM metformin treatment, SWΔFer/FerT cells were profoundly affected by metformin and their viability was significantly impaired after 24 h of treatment with 10-mM metformin (Figure 1B). Annexin V/PI staining analysis revealed that the main form of death induced by metformin was primarily late apoptosis (Figure 1C).

To examine whether metformin affects mitochondrial function in the treated metastatic cells, the level of ATP was determined under conditions in which Oxphos prevails (low glucose/high glutamine). This revealed a significant decrease in cellular ATP levels in metformin-treated SWΔFer/FerT cells compared to metformin-treated parental SW620 cells (Figure 1D). Since metformin was shown to inhibit the activity of the ETC Comp. I type I NADH dehydrogenase (ubiquinone) [21,22,23] with which Fer and FerT associate in SW620 cells [8], we compared NAD^+^ levels in SW620 and SW620ΔFer/FerT cells. This parameter inversely reflects the mitochondrial Comp. I activity level. While NAD^+^ levels in SW620 cells were barely affected even after 24 h of metformin treatment, NAD^+^ levels were drastically decreased 16 and 24 h posttreatment in SWΔFer/FerT cells (Figure 1E).

**Figure 2:**
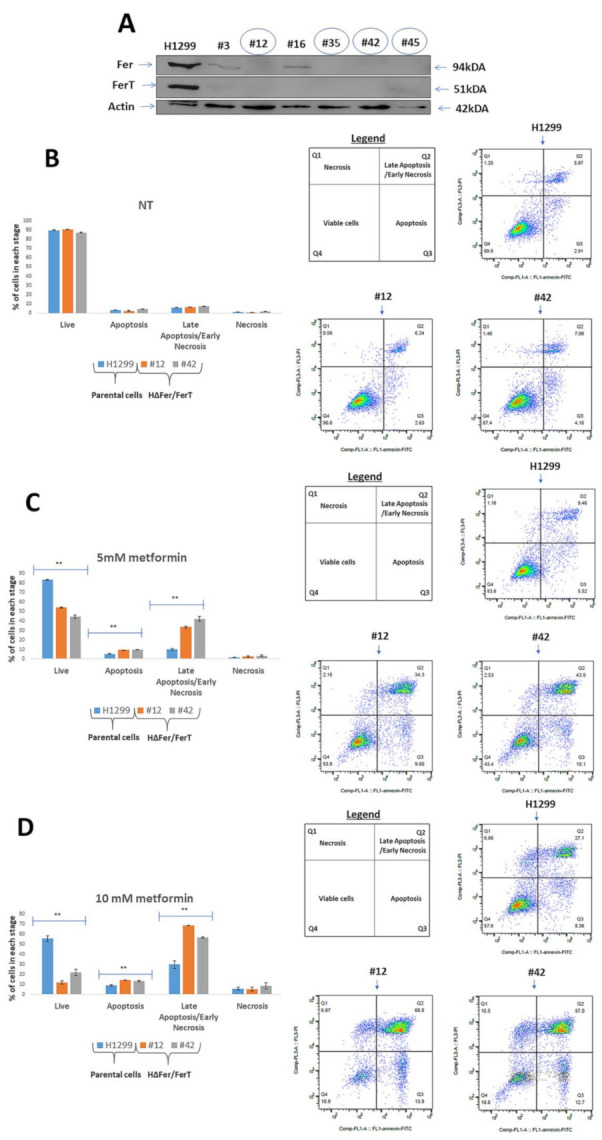
Lack of Fer and FerT renders metastatic lung cancer cells susceptible to metformin treatment. (**A**) Fer- and FerT-deficient HΔFer/FerT cells were generated using the CRISPR-Cas9 system. Western blot analysis of lysates from parental H1299 cells expressing both Fer and FerT proteins, and six derived HΔFer/FerT knock-out clones (#3, #12, #16, #35, #42, and #45) are shown. Actin served as a loading control. (**B**) Parental H1299 and HΔFer/FerT cells were either left untreated (NT) or treated with (**C**) 5-mM metformin or (**D**) 10-mM metformin for 24 h. Cells were stained with annexin V/PI followed by flow-cytometry analysis. Annexin V staining (Q3) indicates apoptotic cell death. PI staining (Q1) indicates necrotic cell death. Annexin V/PI double staining (Q2) indicates late apoptotic/early necrotic cell death. Each experiment represents one out of three independent experiments that gave similar results. ** denotes *p* < 0.01.

To check the generality of our findings, we turned to knocking out the *fer* and *fer*T genes in other colon cancer cell lines. However, these attempts failed in several colon cancer cell lines derived from different stages of the disease (Supplementary Materials Figure S1). Of the other cancer cells lines tested, we managed to knockout the *fer* and *fer*T genes in metastatic NSCLC H1299 cells (Figure 2A). As seen with SWΔFer/FerT cells, Fer- and FerT-deficient H1299 (HΔFer/FerT) cells were significantly more sensitive to metformin treatment than the parental H1299 cells (Figure 2B–D).

### 3.2. FerT Governs the Vulnerability of SW620 Cells to Metformin 

**Figure 3:**
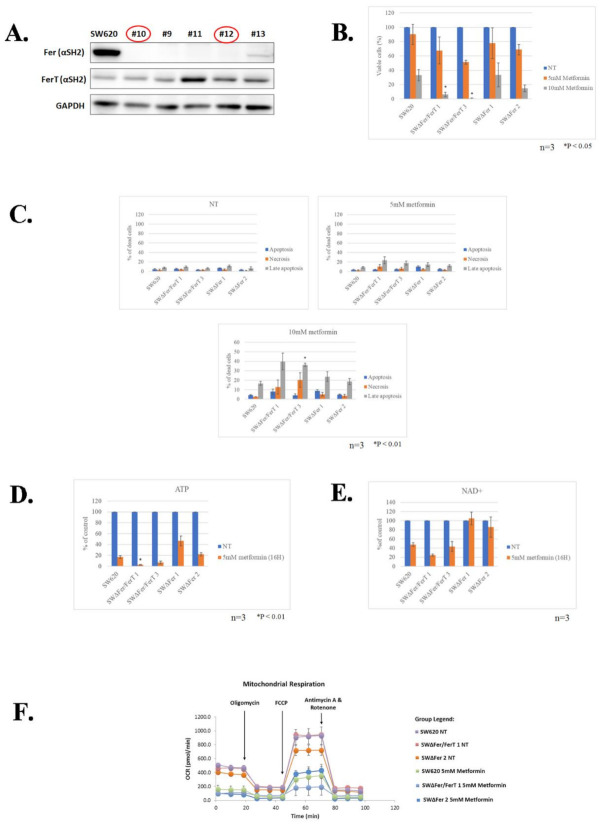
FerT reduces the sensitivity of SW620 cells to metformin. (**A**) SWΔFer clones expressing FerT without Fer were established. The *fer* gene was selectively disrupted in SW620 cells using a CRISPR/Cas9 system. Western blot analysis of lysates from parental SW620 cells and SW620ΔFer knockout clones: clones #10 and #12 (referred to as SWΔFer1 and SWΔFer2, respectively) were selected for further analysis. (**B**)**.** Parental SW620, SWΔFer/FerT, and SWΔFer cells were either left untreated (NT) or treated with metformin for 24 h. Dead and viable cells were differentiated using Trypan Blue staining and counted using Countess II cell-counter. Values represent means ± SE (*n* = 3). (**C**)**.** Cells were stained with annexin V/PI, and viable and dead cell subpopulations were quantified by flow cytometry analysis. Values represent means ± SE (*n* = 3). (**D,E**) Mitochondrial sensitivity to metformin decreased in SWΔFer cells. Cells were either left untreated (NT) or treated with 5-mM metformin for the indicated time periods. (**D**)**.** Cellular ATP levels (there is a significant effect of cell type on percent change of viable cells (1-way ANOVA, F(4, 10) = 23.26, *p* < 0.0001) and (**E**) cellular NAD+ levels of the different clones were measured using HPLC analysis. Values represent means ± SE. (**F**) Mitochondrial respiration was determined in untreated (NT) or treated with 5-mM metformin SW620, SWΔFer/FerT 1, and SWΔFer 2 cells. Each experiment represents one out of three independent experiments that gave similar results.

To discern the roles of Fer and FerT in modulating the sensitivity of SW620 cells to metformin, we selectively knocked-out the *fer* gene while preserving *ferT* and the encoded FerT protein. Multiple SWΔFer clones that failed to express Fer but maintained FerT levels similar to parental SW620 cells were generated, two of which (#10, and #12, henceforth referred to as SWΔFer 1 and SWΔFer 2, respectively) were selected for further studies (Figure 3A).

We then compared the sensitivity of parental SW620, SWΔFer/FerT, and SWΔFer cells to metformin. Unlike SWΔFer/FerT cells (SWΔFer/FerT 1 and 3), which exhibit increased sensitivity to 10 mM metformin, SWΔFer cells (SWΔFer 1 and 2) expressing FerT but not Fer maintained moderate sensitivity to metformin like parental SW620 cells (Figure 3B).

The decreased sensitivity to metformin of SWΔFer cells was reflected by a decreased level of late apoptosis and necrosis induced by metformin in these cells in comparison to SWΔFer/FerT cells (Figure 3C).

**Figure 4:**
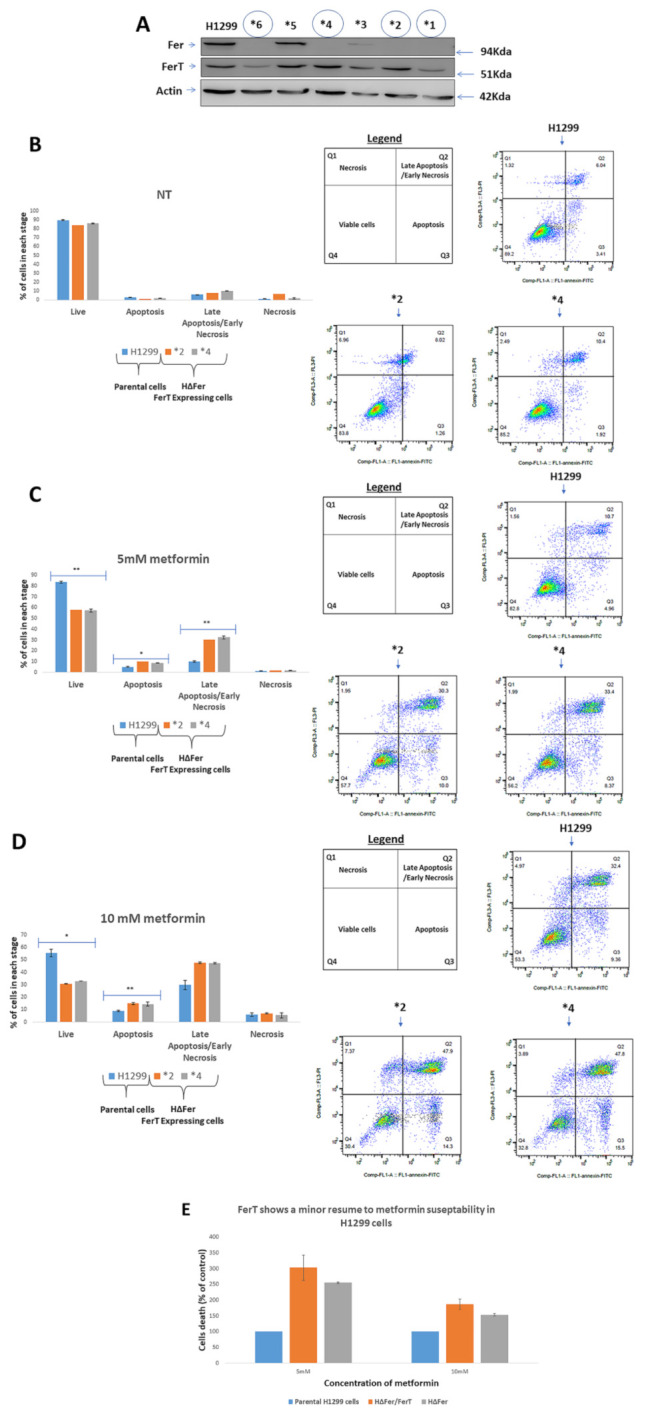
Fer is required for alleviating the susceptibility of H1299 cells to metformin. (**A**) HΔFer clones expressing FerT without Fer were established. The *fer* gene was selectively disrupted in H1299 cells using a CRISPR/Cas9 system. Western blot analysis was performed for lysates from H1299 cells and HΔFer knockout clones. Clones *2 and *4 were selected for further analysis. (**B**) Parental H1299 and HΔFer cells were either left untreated (NT) or treated with (**C**) 5-mM metformin or (**D**) 10-mM metformin for 24 h. Cells were stained with annexin V/PI, and viable and dead cell subpopulations were quantified by flow cytometry analysis. Values represent means ± SE of one out of three independent experiments that gave similar results. (**E**) The relative effects of Fer/FerT deficiency or Fer deficiency alone on H1299-cell sensitivity to metformin: values are means ± SE of percentage of dead cells compared to the parental H1299 cells and represent one out of three independent experiments that gave similar results. * denotes *p* < 0.05, ** denotes *p* < 0.01.

To examine whether the reduced sensitivity of SWΔFer cells to metformin is also reflected by moderate downregulation of mitochondrial functions, we compared cellular ATP levels in SW620, SWΔFer/FerT, and SWΔFer cells. While this revealed a significant decrease in cellular ATP in metformin-treated SWΔFer/FerT cells, the ATP and NAD+ levels in treated SWΔFer cells, which express FerT, were similar to that measured in parental SW620 cells (Figure 3D,E). Coinciding with the above findings, mitochondrial respiratory activity was higher in metformin-treated SWΔFer cells compared with SWΔFer/FerT cells and matched the mitochondrial respiration rate in SW620 cells (Figure 3F). Thus, FerT modulates the sensitivity of SW620 cells to metformin. Notably, unlike SW620 cells, in H1299 cells, the expression of FerT alone (Figure 4A, clones HΔFer, *2, and *4) showed only marginal resumption of the moderate sensitivity of these cells to metformin (Figure 4B–E), suggesting that Fer is required for resuming moderate sensitivity of these cells to metformin.

### 3.3. FerT Governs The Sensitivity of SW620 Cells to Hypoxic Stress

**Figure 5:**
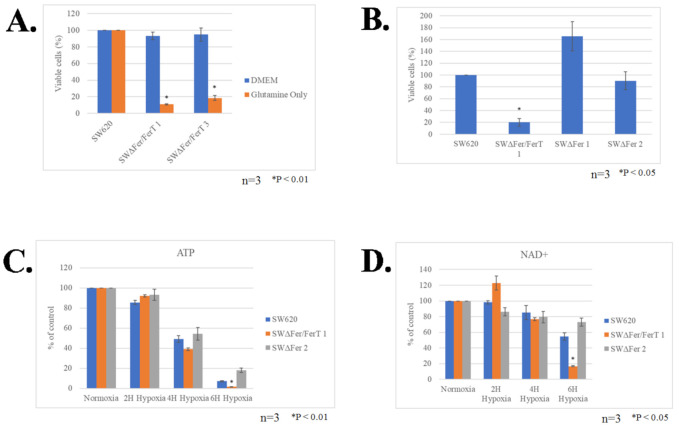
FerT governs the sensitivity of SW620 cells to hypoxic stress. (**A**) Parental SW620 and SWΔFer/FerT cells were grown in the presence (DMEM) or absence (glutamine only) of glucose and were subjected to hypoxia for 24 h. Cells were then harvested, and dead and viable cell fractions were quantified using Trypan Blue staining and Countess II, cell-counter. Values represent means ± SE (*n* = 3). (**B**) Parental SW620, SWΔFer/FerT 1, and SWΔFer 1 and 2 cells were grown in glucose-deprived medium and subjected to hypoxic stress for 24 h. Cells were harvested, and the fractions of viable and dead cells were determined using Trypan Blue staining and Countess II cell-counter. Values represent means ± SE (*n* = 3). There is a significant interaction between treatment and cell type on the percent change of viable cells (2-way ANOVA, F(4, 18) = 53.6, *p* < 0.0001). (**C,D**) FerT restrains mitochondrial sensitivity to hypoxic stress. SW620, SWΔFer/FerT 1, and SWΔFer 2 cells grown in glutamine-supplemented glucose-deprived medium were subjected to hypoxic stress for the indicated time periods. (**C**) Cellular ATP levels and (**D**) Cellular NAD^+^ levels: values represent means ± SE. Each experiment represents one out of three independent experiments that gave similar results.

**Figure 6:**
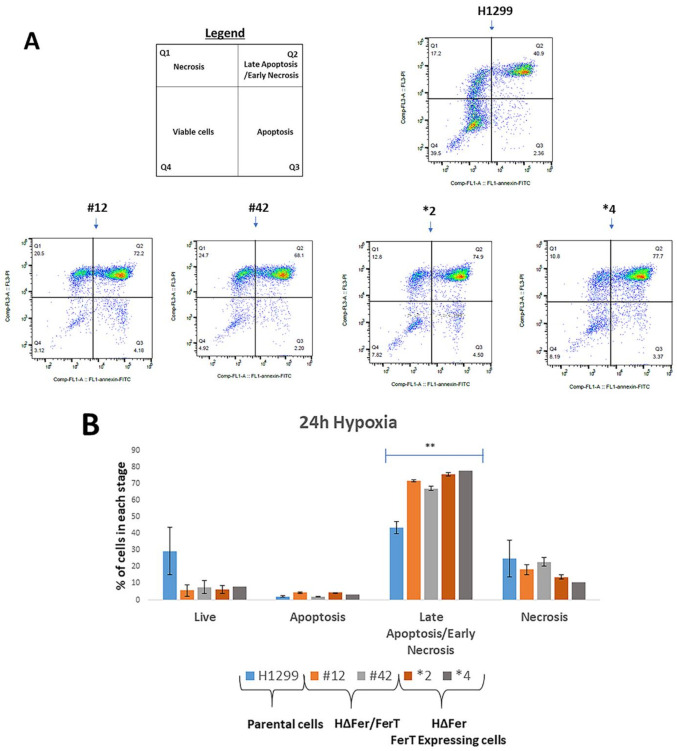
Lack of Fer and FerT renders metastatic H1299 cells susceptible to hypoxic stress. (**A**) Parental H1299, HΔFer/FerT, and HΔFer cells were grown in the absence of glucose (glutamine only) and were subjected to hypoxia for 24 h. Cells were stained with annexin V/PI followed by flow-cytometry analysis. (**B**) Quantification of the results presented in (**A**): values represent means ± SE of one out of three independent assays that gave similar results. ** denotes *p* < 0.01.

The modulatory role of FerT on the sensitivity of SW620 cells to metformin prompted us to examine the role of this cancer-specific Fer variant in governing malignant-cell sensitivity to hypoxia, another stress affecting mitochondria in solid tumors. SW620 and SWΔFer/FerT cells were grown in either glucose-reach or glucose-deprived medium supplemented with glutamine to promote glycolysis or mitochondrial Oxphos, respectively. The cells were subjected to hypoxic stress for 24 h, after which their viability was determined. While no obvious difference between the survival of SW620 and SWΔFer/FerT cells could be seen under high glucose (DMEM) (Figure 5A), glucose deprivation (glutamine only) of SWΔFer/FerT cells rendered them significantly more susceptible to hypoxia (Figure 5A). Notably, the expression of FerT alone in the absence of Fer, endowed the SWΔFer cells, with minor sensitivity to hypoxia exhibited by the parental SW620 cells (Figure 5B). Accordingly, FerT maintained moderate mitochondrial sensitivity to hypoxic stress as reflected by the similar ATP and NAD^+^ levels measured in hypoxic SWΔFer and the parental SW620 cells (Figure 5C,D). As was seen for SWΔFer/FerT cells, Fer- and FerT-deficient H1299 cells (HΔFer/FerT) exhibited increased sensitivity to hypoxic stress. However, unlike the SW620 cells, the expression of FerT alone did not resume moderate sensitivity to hypoxia exhibited by the parental H1299 cells (Figure 6A,B).

Hence, while FerT governs the sensitivity of SW620 cells to hypoxic stress, the presence of Fer is required for maintaining the moderate mitochondrial susceptibility of H1299 cells to hypoxic cue.

## 4. Discussion

Several clinical studies have shown that metformin can attenuate the proliferation and growth of breast, prostate, and endometrial cancer or malignant tumors [33,34,35,36,37]. However, even patients that initially respond to metformin acquire resistance to this drug [38]. Thus, there is an eminent need to extend our understanding of the molecular pathways that promote resistance to metformin. A pharmacodynamics window study of metformin in breast cancer patients revealed that tumors with upregulated Oxphos genes showed impaired response to metformin [38]. These findings imply and substantiate the notion that upregulation of the mitochondrial Oxphos could reduce the sensitivity of cancer cells to metformin [7]. This could coincide with the observation that one of the primary targets of metformin anticancer activity is inhibition of mitochondrial Comp. I function [21]. Furthermore, a recent work by Liu et al. comparing the metabolite profile of ten ovarian tumor samples from untreated and ten metformin-treated patients demonstrated decreases in the levels of some TCA cycle intermediates [39]. Hence, metformin interferes also with mitochondria functioning in vivo, thereby restricting the survival of cancer cells. Upregulation of Oxphos through Oxphos gene induction or the reprogramming of mitochondrial ETC in malignant cells could therefore endow cancer cells with reduced sensitivity and acquired resistance to the metformin growth suppressive effects [7,40]. In the current work, we showed that the intracellular tyrosine kinase Fer and its cancer-specific variant, FerT, which associate with Comp. I and potentiate its activity in malignant cells under stress-imposed conditions [8], play this alleviating role. The absence of the two enzymes is shown to increase mitochondrial vulnerability and susceptibility of metastatic cancer cells to metformin. It should be noted that the metastatic cell lines studied in the current work are insensitive to low glucose growth conditions (Supplementary Materials Figure S2), and their moderate susceptibility to metformin coincides with a previous study indicating that insensitivity to low glucose is linked to a low sensitivity of cancer cells to metformin [7]. Thus, our findings define Fer and FerT as novel supporters of the impaired susceptibility of low-glucose insensitive metastatic cells, to metformin treatment.

The recruitment of Fer and the sperm- and cancer-specific FerT to the reprogrammed ETC Comp. I of malignant cells seems to also enable mitochondria functioning under metabolic stress conditions encountered by abnormally growing cancer cells. One such metabolic challenge is hypoxic stress evoked in solid tumors, which outgrow their vasculature, and in metastatic cells that detach from the primary tumor and begin a dissemination process in the patient’s body [41,42]. The fact that Fer and FerT decrease the susceptibility of metastatic malignant cells to hypoxic stress may have translational ramifications on the development of new anti-metastatic therapeutic approaches directed toward Fer and FerT. Of note is the fact that, while in SW620 cells FerT alone could direct reduced susceptibility of the cells to hypoxia and metformin, in the H1299 NSCLC cells, FerT alone did not have this significant impact. Thus, in H1299 cells, either Fer may have a dominant modulatory role on cell sensitivity to hypoxia and metformin or a collaborative functioning of Fer and FerT is required for exerting their susceptibility-alleviating effects on these cells. Interestingly, H1299 clones lacking both Fer and FerT, or Fer alone exhibited similar mitochondrial activity (Supplementary Materials Figure S3). While our study does not exclude the possibility that Comp. I-associated Fer can also decrease the sensitivity of SW620-cell mitochondria to metformin and hypoxia, our findings do indicate that the cross-regulatory roles of Fer and FerT may differ among distinct metastatic cell types. However, since Fer and FerT share a common kinase domain [8], targeting the kinase domain of the two enzymes with a synthetic molecule should increase the sensitivity of cancer cells to hypoxia and biguanides. Late-phase clinical trials investigating metformin as an anticancer drug are underway. Combining metformin with a specific Fer/FerT inhibitor may potentiate the therapeutic efficacy of this commonly used antidiabetic and anticancer compound. 

## Data Availability

Data sharing not applicable

## Supplementary Materials

The following are available online at https://www.mdpi.com/2073-4409/10/1/97/s1, Figure S1: Colon cell lines derived from different stages of the disease failed to lose the expression of *fer* and *ferT* genes. Figure S2: SW620 and H1299 cells are insensitive to low-glucose growth conditions. Figure S3: Similarly impaired mitochondrial activity in HΔFer/FerT and HΔFer clones.
