# Supplementary material for: Fer and FerT Govern Mitochondrial Susceptibility to Metformin and Hypoxic Stress in Colon and Lung Carcinoma Cells
Source: Cells. 2021 Jan 7;10(1):97. doi: 10.3390/cells10010097 (PMC7826929; doi:10.3390/cells10010097)

Report for Cell Line Authentication

The Cell line authentication test was performed at the Genomics Center of Biomedical Core Facility, Technion.

The test was performed using the Promega GenePrint 24 System in order to determine short tandem repeat (STR) profile of 23 loci plus Amelogenin for gender determination (X or XY). In addition, the male-specific DYS391 locus is included to identify null Y allele results for Amelogenin. DNA sample from the kit (2800M Control DNA) was included in the analysis and served as positive control for the PCR step. No DNA template was also included as negative control.

The results were analyzed using the 3500xl Genetic Analyzer (Life Technologies) and GeneMapper IDX software. Allelic ladder was included in the run. Importantly, the 3500xl system is calibrated once a year and spectral calibrated once every 3 months by authorized person.

This report includes the STR results obtained for your cell line together with the available STR profile. It is important to note that not all STRs that are included in the kit are published. Usually, we compare the results to the Cellosaurus, a cell-line knowledge resource (Bairoch A., J. Biomol Tech, 2018).

The interpretation of results is based on allele calls for all 24 loci and should aid determine whether your cell line is indeed the cell line in question or has profile characteristics which indicate a change, contamination or misidentification.

The raw data appears in Appendix A.

Results

Cell line ID: NCI-H1299 (Cellosaurus no. CVCL_0060)

Marker	NCI-H1299	Cellosaurus
**AMEL**	X	X
**D3S1358**	17	17
**D1S1656**	12,15	
**D2S441**	11,13	
**D10S1248**	13,17	
**D13S317**	12	12
**Penta E**	11	11
**D16S539**	12,13	12,13
**D18S51**	16	16
**D2S1338**	23,24	
**CSF1PO**	12	12
**Penta D**	13	13
**TH01**	6,9.3	6,9.3
**vWA**	16,18	16,18
**D21S11**	32.2	32.2
**D7S820**	10	10
**D5S818**	11	11
**TPOX**	8	8
**DYS391**	-	
**D8S1179**	10,13	10,13
**D12S391**	21,22	
**D19S433**	14	
**FGA**	20	20
**D22S1045**	15,16	

Summary

The sample profile matches 16 out of the 16 available loci of the STR profile from Cellosaurus for line NCI-H1299 (CVCL_0060).
In light of these results, it seems that the tested cell line is indeed NCI-H1299.

Date: 15.7.19

Doron Fogel, MSc, Application specialist **Signature:** ______________

Liat Linde, PhD, Head **Signature:** ______________

We would appreciate the acknowledgment of the Genomics Center of Biomedical Core Facility, Technion in your published work that utilizes the cell line above.

Appendix A

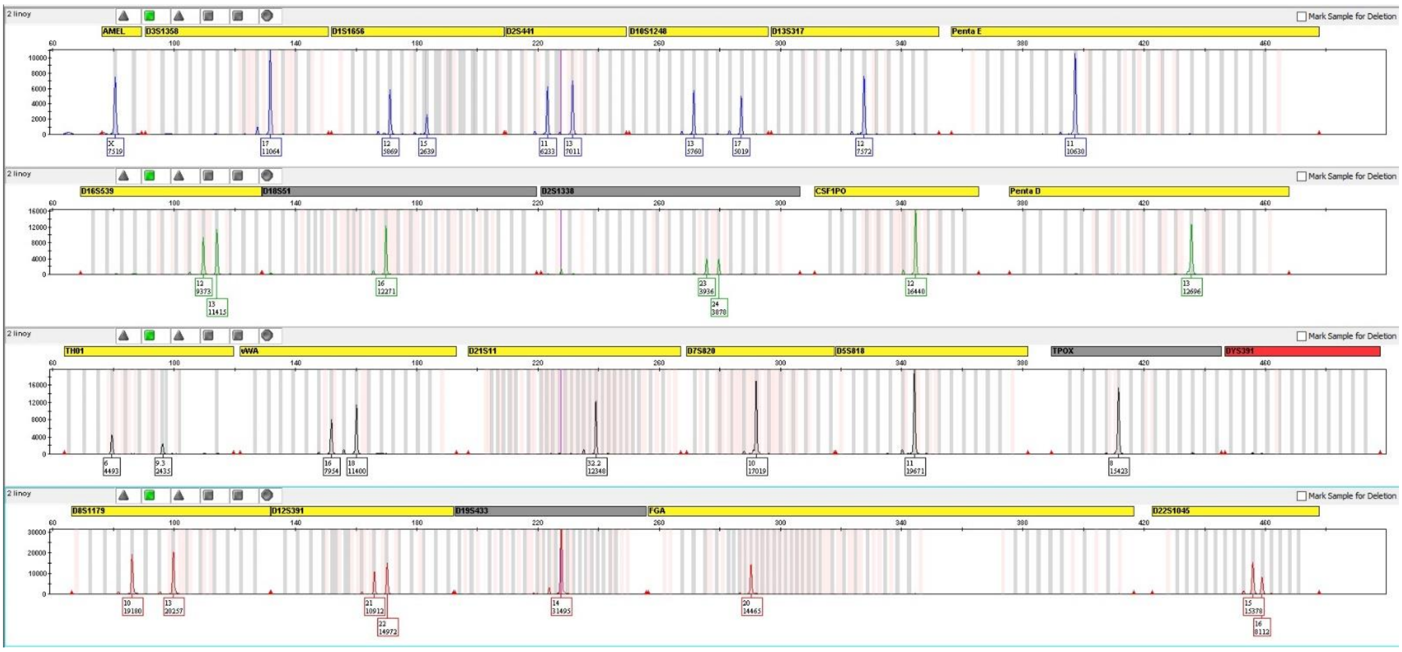

Supplement: Supplementary file 1 [file cells-10-00097-s001.zip › H1299 parental -Authentification.pdf]
