# Supplementary material for: Fer and FerT Govern Mitochondrial Susceptibility to Metformin and Hypoxic Stress in Colon and Lung Carcinoma Cells
Source: Cells. 2021 Jan 7;10(1):97. doi: 10.3390/cells10010097 (PMC7826929; doi:10.3390/cells10010097)
Supplement: Supplementary file 1 [file cells-10-00097-s001.zip › Supplementary Figures.pdf]

Supplementary Figures

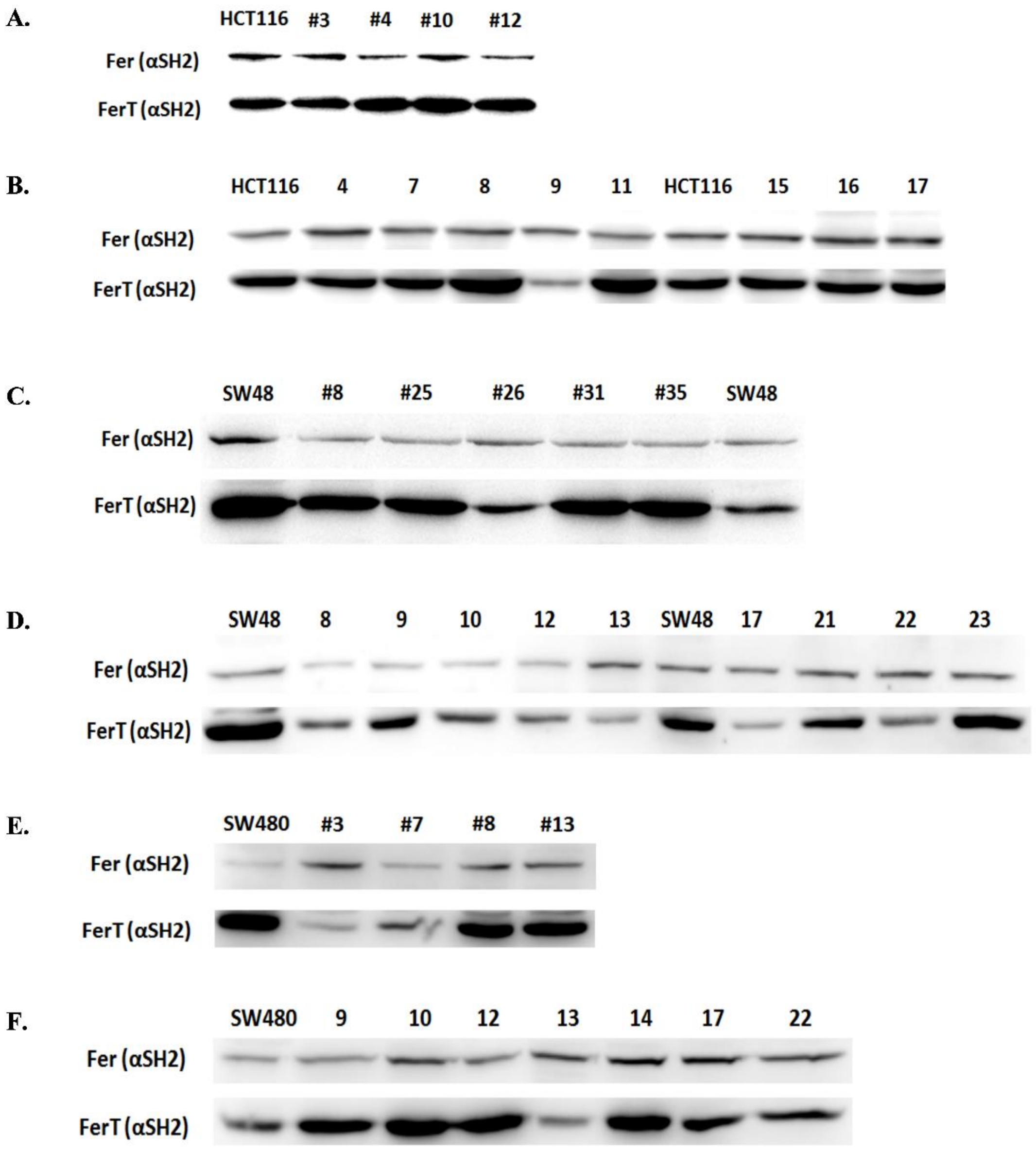

Fig S1. Colon cell lines derived from different stages of the disease failed to lose expression of *fer* and *ferT* genes. CRISPR-Cas9 system failed to knock-out *fer* and *ferT* (upper blots) or *fer* alone (lower blots) in [A-B] HCT116 cells, [C-D] SW48 cells, and [E-F] SW480 cells.

A.

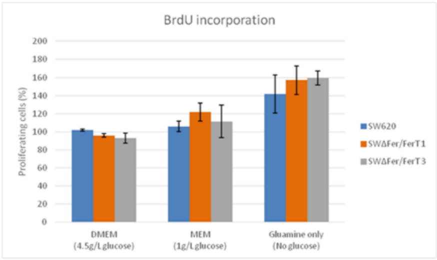

B.

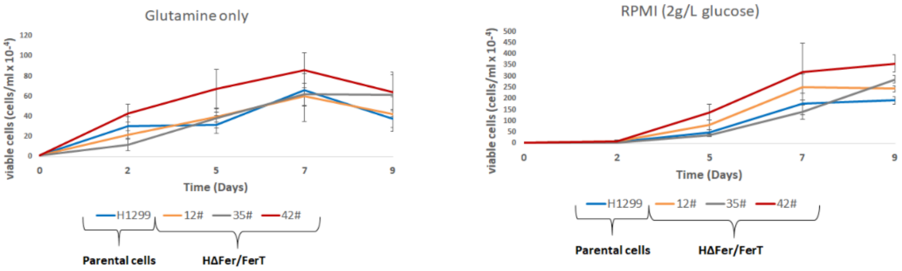

C.

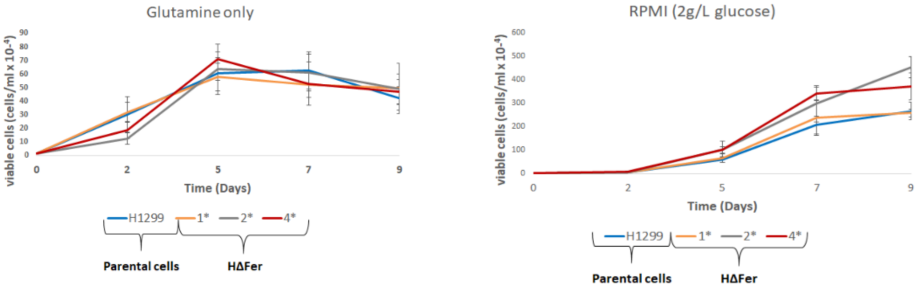

Fig S2. SW620 and H1299 cells are insensitive to low- glucose growth conditions. A, SW620 cells were grown for 48 hours in different media, as indicated. BrdU incorporation levels were followed for additional 24 hours, according to the manufacturer's instructions (BrdU Cell Proliferation Kit, Biovision). Values represent means ±SE. **B-C.** H1299 cells were grown in (**B**) RPMI or (**C**) Glutamine only containing medium for the indicated time periods. Dead and viable cells were differentiated using Trypan Blue staining and were counted using the Countess II cell-counter. Values represent means ±SD.

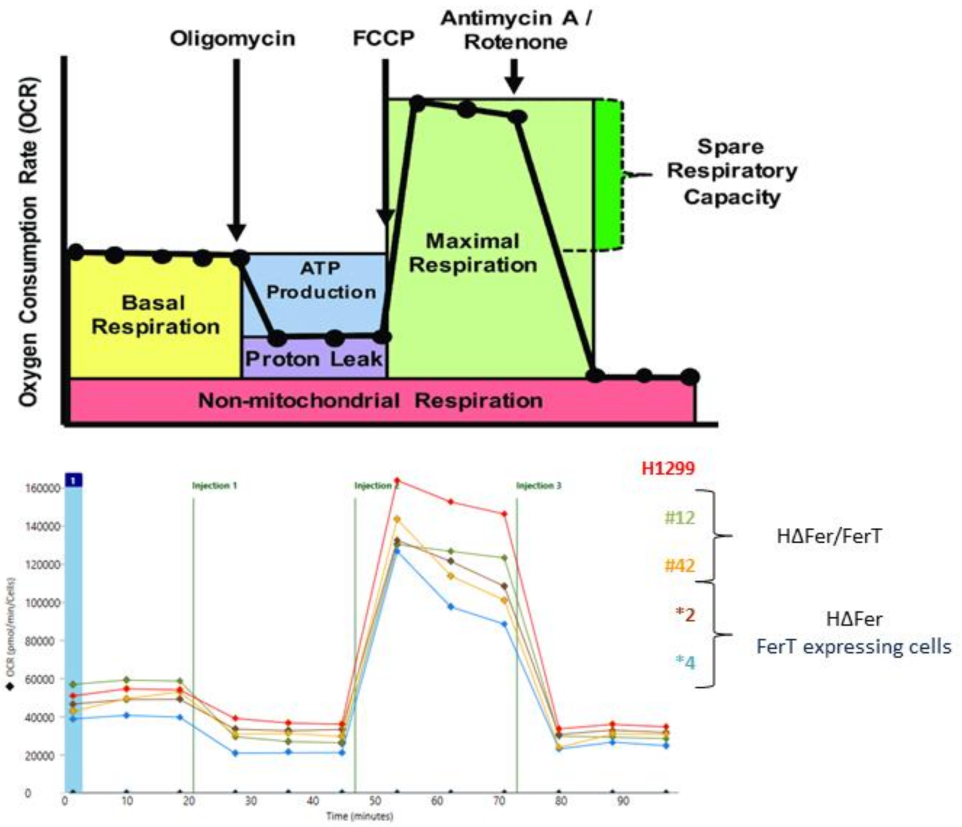

Figure S3. Similarly impaired mitochondrial activity in HΔFer/FerT and HΔFer clones. Mitochondrial respiration was determined in untreated (NT) parental H1299, HΔFer/FerT (#12,#42) and HΔFer (*2,*4) cells.
